# Another Medical School

**Published:** 1846-06

**Authors:** 


ARTICLE XII.

ANOTHER MEDICAL SCHOOL.

A new Medical College is now being established at Memphis. Tennessee. So far as organised, the following appointments have been made by the Trustees.

J. M. BYBEE, M. D., *Professor of Anatomy.*

D. J. M. DOYLE, M. D., *Professor of Surgery.*

A. HOPTON, M. D., *Professor of Chemistry and Pharmacy.*

G. R. GRANT, M. D., *Professor of Theory and Practice.*

The Trustees invite applications for the chairs of Institutes and Medical Jurisprudence, Materia Medica, and Obstetrics. Applications to be sent previous to the first of July next, to R. H. Patillo, Secretary of the Board. W. B. H.

